# Validity of self-reported educational level in the Tromsø Study

**DOI:** 10.1177/14034948221088004

**Published:** 2022-05-20

**Authors:** Chi Q Vo, Per-Jostein Samuelsen, Hilde L Sommerseth, Torbjørn Wisløff, Tom Wilsgaard, Anne E Eggen

**Affiliations:** 1 Department of Community Medicine, UiT The Arctic University of Norway, Tromsø, Norway; 2 Regional Medicines Information and Pharmacovigilance Centre (RELIS), University Hospital of North Norway, Tromsø, Norway; 3 The Norwegian Historical Data Centre, UiT The Arctic University of Norway, Tromsø, Norway

**Keywords:** Self-report, education, validation, survey, completeness, correctness

## Abstract

**Background:**

Self-reported data on educational level have been collected for decades in the Tromsø Study, but their validity has yet to be established.

**Aim:**

To investigate the completeness and correctness of self-reported educational level in the Tromsø Study, using data from Statistics Norway. In addition, we explored the consequence of using these two data sources on educational trends in cardiometabolic diseases.

**Methods:**

We compared self-reported and Statistics Norway-recorded educational level (primary, upper secondary, college/university <4 years, and college/university ⩾4 years) among 20,615 participants in the seventh survey of the Tromsø Study (Tromsø7, 2015–2016). Sensitivity, positive predictive value and weighted kappa were used to measure the validity of self-reported educational level in three age groups (40–52, 53–62, 63–99 years). Multivariable logistic regression was used to compare educational trends in cardiometabolic diseases between self-reported and Statistics Norway-recorded educational level.

**Results:**

Sensitivity of self-reported educational level was highest among those with a college/university education of 4 years or more (⩾97% in all age groups and both sexes). Sensitivity for primary educational level ranged from 67% to 92% (all age groups and both sexes). The lowest positive predictive value was observed among women with a college/university education of 4 years or more (29–46%). Weighted kappa was substantial (0.52–0.59) among men and moderate to substantial (0.41–0.51) among women. Educational trends in the risk of cardiometabolic diseases were less pronounced when self-reported educational level was used.

**Conclusions:**

**Self-reported educational level in Tromsø7 is adequately complete and correct. Self-reported data may produce weaker associations between educational level and cardiometabolic diseases than registry-based data.**

## Introduction

Self-administrated questionnaires are often used in epidemiological studies to obtain information about a person’s education. Inaccurate self-reported data occur when individuals answer questions incorrectly, which can lead to exposure misclassification, and thereby to less reliable study findings [1]. Education is an important determinant of socioeconomic status, as it confers skills that help individuals utilise health information, and it affects future income and occupational class [2, 3]. Indeed, education has become the principal pathway to higher incomes, stable employment and healthier lifestyle [4]. Furthermore, as self-reported education is often used as an exposure and covariate in health research [5, 6], it is important to assess the validity of that variable. Validation studies on this variable should be done to produce estimates of misclassification in self-reported data and help determine if study results are biased. Data accuracy can be determined by comparing self-reported data to a gold standard data source and is often calculated by two measures: correctness, the proportion of recorded observations in the registry that are correct; and completeness, which measures the proportion of recorded observations that are actually recorded in the gold standard data source [7]. Studies of the quality of reported education are not new in the literature. However, research on the validity of self-reported education within epidemiology is still scarce. This study aimed to investigate the completeness and correctness of self-reported educational level in the Tromsø Study, using data from Statistics Norway (SSB). In addition, we explored the consequence of using these two data sources on educational trends in cardiometabolic diseases.

## Methods

### The Tromsø Study

The Tromsø Study is an ongoing population-based health survey, which consists of seven surveys (Tromsø1–7) conducted between 1974 and 2016 in the municipality of Tromsø, northern Norway. The study population consists of complete birth cohorts and random samples of other cohorts [8, 9]. All inhabitants of the municipality aged 40 years and above were invited to participate in Tromsø7 (2015–2016), and the study questionnaire collected information on topics such as health issues, symptoms, diseases, use of medication and healthcare services, employment, and sociodemographic and lifestyle factors.

### Study population

Data on self-reported educational level from Tromsø7 was linked to data from SSB, the national statistical institute of Norway and the main producer of official statistics, using the unique 11-digit identification number assigned to all individuals living in Norway. A total of 21,083 people participated in Tromsø7 (attendance 65%), of which 20,615 had records in SSB and were included in the analyses. A total of 468 were excluded from the analysis. Of these 99 persons lack information about education in SSB (19 persons were specified as ‘no education, unspecified, and preschool education’) and 369 had no education in Tromsø7.

### Self-reported educational level, household income, and other variables

In the Tromsø7 questionnaire, participants were asked to respond to the question: ‘What is the highest level of education you have completed?’. Response options were: primary/partly secondary education (up to 10 years of schooling); upper secondary education (minimum 3 years); tertiary education, short: college/university less than 4 years; and tertiary education, long: college/university 4 years or more (see link to questionnaire in Supplemental material). They were also asked to report their total pre-taxable household income for the previous year, using eight categories from 150,000 NOK or less to 1,000,000 NOK or more. The two lowest income groups (⩽150,000 NOK and 150,000–250,000 NOK) were merged in the analysis. Participants reported their current and previous status for the following cardiometabolic diseases: diabetes, myocardial infarction, angina pectoris, and cerebral stroke, which were categorised as binary variables. Participants reported their self-rated health status as ‘very bad’, ‘bad’, ‘neither good nor bad’, ‘good’ and ‘excellent’, which was regrouped into three categories (‘bad’, ‘neither good nor bad’ and ‘good’). Finally, participants reported whether or not they lived with a spouse.

### SSB-recorded educational level

Educational information in SSB comes from administrative sources, such as educational institutions, and the State Educational Loan Fund provides supplemental data on education acquired abroad [10]. SSB records the highest completed educational level. The Norwegian Standard Classification of Education has nine educational levels alone, including a value for unspecified level [11]. These were regrouped by SSB into: no education or preschool education; primary education; upper secondary education; vocational education; university/college education, short; and university/college education, long. We furthermore excluded participants in the group with no education, preschool education or unspecified education from the analysis. We also merged the categories upper secondary education and vocational education leaving four educational levels (primary education, upper secondary education, university/college education <4 years, and university/college education ⩾4 years) that were comparable to the self-reported educational levels in Tromsø7.

### Statistical analyses

We assessed the validity of self-reported educational level in Tromsø7 by estimating sensitivity (completeness) and positive predictive value (PPV, correctness), using SSB-recorded educational level as the gold standard. Agreement between self-reported and SSB-recorded educational level was measured by percentage observed agreement and weighted kappa. Kappa values and kappa agreement were interpreted as proposed by Viera and Garrett [12] (less than chance: <0.00, slight: 0.00–0.20, fair: 0.21–0.40, moderate: 0.41–0.60, substantial: 0.61–0.80, or almost perfect: 0.81–1.00). Multinomial logistic regression was used to calculate odds ratios (ORs) of over or underreporting educational level. Comparisons between self-reported and SSB-recorded educational level were stratified by age group (40–52, 53–62 and 63–99 years) and sex. These age groups were constructed after taking into account the school reform of 1959, when 7 years of primary education was made mandatory. Those who started primary school in 1959 were 63 years old in Tromsø7. The 53–62 age group was constructed to reflect another school reform in 1969. Logistic regression models were also used to estimate ORs of self-reported cardiometabolic diseases in Tromsø7 according to self-reported and SSB-recorded educational levels. A randomisation test with 10,000 permutations of the data file was used to compare trends, that is, the categorical educational level variable modelled as a linear term, between self-reported and SSB-recorded educational level. The linearity assumption was reasonably met and self-reported and SSB-recorded educational levels were therefore modelled as linear terms.

### Ethics

This study was approved by the Norwegian Centre for Research Data (NSD Data Protection Services) (reference 809230). All participants in the Tromsø Study have given written informed consent for their data to be used in research. This study was not defined as health research by the Regional Ethics Committee North and was exempted from the requirement of study preapproval.

## Results

Of the 20,615 individuals included in the analysis, 53% were women; the mean age was 57 years (standard deviation (SD): 11.3 years, range: 40–99 years). The proportion of women with college/university education of 4 years or more was higher than that among men (33% vs. 26%, respectively); this was also seen for the primary educational level (24% vs. 22%, respectively). The proportion of women with household income of 1,000,000 NOK or more was lower than that among men (22% vs. 28%, respectively) (Table I).

**Table I.** Socioeconomic characteristcs of study population in the Tromsø Study 2015–16.

	Women (%) *n*=10,826	Men (%) *n*=9789
Age group		
40–52 years	4372 (41.4)	3865 (39.5)
53–62 years	3067 (28.3)	2682 (27.4)
63–99 years	3387 (31.3)	3242 (33.1)
Educational level		
Primary education	2597 (24.0)	2163 (22.1)
Upper secondary education	2749 (25.4)	2989 (30.5)
College/university <4 years	1913 (17.7)	2082 (21.3)
College/university ⩾4 years	3567 (32.9)	2555 (26.1)
Household income^a,b^		
<250,000 NOK	725 (7.0)	396 (4.1)
251,000–350,000 NOK	892 (8.7)	509 (5.3)
350,000–450,000 NOK	1110 (10.8)	764 (7.9)
450,000–550,000 NOK	1311 (12.7)	976 (10.1)
550,000–750,000 NOK	1749 (17.0)	1780 (18.5)
750,000–1,000,000 NOK	2259 (22.0)	2453 (25.5)
⩾1,000,000 NOK	2244 (21.8)	2744 (28.5)

Values are numbers (%).

a 100,000 NOK ≈ 11,500 USD.

b 703 missing value.

Sensitivity of self-reported educational level was highest among those with a college/university education of 4 years or more (⩾97% in all age groups and both sexes), and lowest among those with a college/university education of less than 4 years (37–58% in all age groups and both sexes) (Table II). Among women who self-reported primary educational level, sensitivity ranged from 67% to 92%, compared to 72–91% among men. PPVs for women with a college/university education of 4 years or more were between 29–46% and 59–62% for men. The PPV was 48–67% among women, compared to 52–66% among men with primary education. In all age groups and both sexes, the highest degree of underreporting in Tromsø7 was observed among those with SSB-recorded upper secondary educational level, but a self-reported primary educational level, whereas the highest degree of overreporting was observed among those with SSB-recorded college/university education less than 4 years, but a self-reported college/university education of 4 years or more (Supplemental Table I and Table II). For women, kappa agreement varied from moderate to substantial (57–64%), and was substantial in all age groups for men (65–71%). A fair corresponding weighted kappa value was found in all age groups for women (0.41, 0.48 and 0.51, respectively), and for men (0.52, 0.54 and 0.59, respectively).

**Table II.** Validity of self-reported educational level compared to that recorded in Statistics Norway by age and stratified by sex. The Tromsø Study 2015–2016.

Age	Women		Men	
	Sensitivity (%)	PPV (%)	Sensitivity (%)	PPV (%)
40–52 years				
Primary education	67.2	66.8	72.3	65.8
Upper secondary education	73.6	86.2	69.0	87.3
College/university <4 years	40.2	77.2	51.3	64.3
College/university ⩾4 years	99.1	46.0	99.1	60.3
53–62 years				
Primary education	70.5	62.8	75.5	61.4
Upper secondary education	64.5	82.2	60.1	86.1
College/university <4 years	37.0	66.4	53.9	54.0
College/university ⩾4 years	98.4	37.5	97.2	58.5
63–99 years				
Primary education	92.0	48.4	90.8	51.5
Upper secondary education	43.3	88.1	49.7	88.6
College/university <4 years	37.2	68.3	57.9	56.5
College/university ⩾4 years	96.7	29.1	96.6	62.1

PPV: positive predictive value.

Among those aged 40–52 and 53–62 years, the proportions of self-reported and SSB-recorded primary educational level were similar. However, in those aged 63–99 years, there was a notable difference (Supplemental Table III). All age groups showed higher self-reported than SSB-recorded college/university education of 4 years or more, and this was especially evident in the youngest age group.

**Figure 1.**
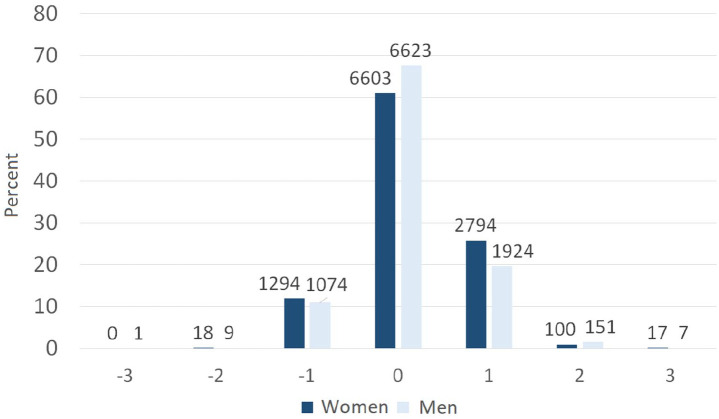
Differences between self-reported and Statistics Norway-recorded educational level by sex. The Tromsø Study 2015–16. Negative numbers indicate underreporting and the positive numbers indicate overreporting.

The difference between self-reported and SSB-recorded educational levels varied by sex (Figure 1), that is, levels of education registered by SSB subtracted self-reported level of education in Tromsø7. Zero represents individuals who self-reported the same educational level as in the SSB registry. Numbers ±1, 2 and 3 indicate levels of underreporting or overreporting. Women were more likely to overreport (OR 1.46, 95% confidence interval (CI) 1.36–1.57) and underreport (OR 1.10, 95% CI 0.99–1.21) their educational level compared to men. Women aged 53–62 years overreported more often than those aged 40–52 years (OR 1.13, 95% CI 1.02–1.26), and the odds of underreporting are higher among women aged 53–62 years (OR 1.95, 95% CI 1.58–2.41) and 63–99 years (OR 4.47, 95% CI 3.68–5.43) compared to 40–49 years (Table III). Higher odds of underreporting was also found among men aged 53–62 years (OR 1.47, 95% CI 1.10–1.94) and 63–99 years (OR 1.29, 95% CI 1.11–1.50) compared to 40–49 years. Men aged 53–62 years overreported more than those aged 40–52 years (OR 1.06, 95% CI 0.94–1.20). For participants who lived with a spouse, the ORs for overreporting were 0.56 (95% CI 0.48–0.64) for women, and 0.73 (95% CI 0.63–0.86) for men. The ORs for underreporting were 1.62 (95% CI 1.36–1.92) for women and 1.46 (95% CI 1.21–1.77) for men. Underreporting educational level was more common among men with bad (OR 1.46, 95% CI 1.10–1.94) and neither good nor bad health (OR 1.29, 95% CI 1.11–1.50), compared with those with good health. Finally, overreporting of educational level increased, while underreporting decreased, with increasing household income.

**Table III.** Sex-specific odds ratios of under and overreporting of educational level from Tromsø7 and Statistics Norway.

	*n* (%)	Overreporting vs. correctly reported OR (95% CI)	Underreporting vs. correctly reported OR (95% CI)
Women			
Age group			
40–52 years	3977 (41.7)	Reference group	Reference group
53–62 years	2767 (29.1)	1.13 (1.02–1.26)	1.95 (1.58–2.41)
63–99 years	2784 (29.2)	0.97 (0.85–1.11)	4.47 (3.68–5.43)
Living with spouse	6926 (72.7)	0.56 (0.48–0.64)	1.62 (1.36–1.92)
Self-rated health			
Bad	575 (6.0)	1.05 (0.85–1.29)	0.94 (0.71–1.26)
Neither good nor bad	2364 (24.8)	0.87 (0.77–0.98)	1.05 (0.90–1.22)
Good	6589 (69.2)	Reference group	Reference group
Household income			
<250,000 NOK	606 (6.4)	0.17 (0.12–0.22)	5.71 (3.92–8.33)
251,000–350,000 NOK	754 (7.9)	0.29 (0.23–0.38)	7.25 (5.07–10.38)
351,000–450,000 NOK	945 (9.9)	0.35 (0.28–0.44)	6.75 (4.79–9.52)
451,000–550,000 NOK	1155 (12.1)	0.65 (0.55–0.78)	4.54 (3.23–6.37)
551,000–750,000 NOK	1635 (17.1)	0.52 (0.45–0.61)	3.40 (2.46–4.69)
751,000–1,000,000 NOK	2217 (23.3)	0.76 (0.67–0.87)	2.40 (1.74–3.33)
⩾1,000,000 NOK	2216 (23.3)	Reference group	Reference group
Men			
Age group			
40–52 years	3702 (39.8)	Reference group	Reference group
53–62 years	2576 (27.7)	1.06 (0.94–1.20)	1.47 (1.10–1.94)
63–99 years	3024 (32.5)	0.98 (0.86–1.12)	1.29 (1.11–1.50)
Living with spouse	7621 (81.9)	0.73 (0.63–0.86)	1.46 (1.21–1.77)
Self-rated health			
Bad	429 (4.6)	1.37 (1.07–1.75)	1.46 (1.10–1.94)
Neither good nor bad	2394 (25.7)	0.93 (0.82–1.05)	1.29 (1.11–1.50)
Good	6479 (69.6)	Reference group	Reference group
Household income			
<250,000 NOK	341 (3.7)	0.29 (0.20–0.44)	6.38 (4.47–9.10)
251,000–350,000 NOK	466 (5.0)	0.41 (0.30–0.56)	5.18 (3.70–7.25)
351,000–450,000 NOK	715 (7.7)	0.52 (0.41–0.67)	4.50 (3.32–6.09)
451,000–550,000 NOK	923 (9.9)	0.64 (0.52–0.79)	4.80 (3.62–6.36)
551,000–750,000 NOK	1717 (18.5)	0.73 (0.62–0.85)	3.20 (2.47–4.14)
751,000–1,000,000 NOK	2417 (26.0)	0.85 (0.75–0.97)	2.26 (1.76–2.91)
⩾1,000,000 NOK	2723 (29.3)	Reference group	Reference group

CI: confidence interval; OR: odds ratio. The Tromsø Study 2015–16.

100,000 NOK ≈ 11,500 USD.

Mutually adjusted for all listed variables.

Total missing values for women *n*=1298.

Total missing values for men *n*=486.

We found educational trends in the risk of self-reported cardiometabolic diseases when using both self-reported and SSB-recorded educational level (Table IV). For women the odds for diabetes increased by 31% per one-level decrease in self-reported educational level (OR 1.31, 95% CI 1.20–1.42), while the odds increased by 44% per one-level decrease in SSB-recorded educational level (OR 1.44, 95% CI 1.29–1.61). We saw the same trends for myocardial infarction, angina pectoris, and stroke, also for men. However, the educational trend was less pronounced when using the self-reported educational level.

**Table IV.** Age-adjusted odds ratios for the association between cardiometabolic diseases and educational level from Tromsø7 and Statistics Norway.

	Tromsø7 OR (95% CI)^a^	Statistics Norway OR (95% CI)^a^	*P* value equality^b^
Women			
Diabetes mellitus (509 out of 10,510)^c^	1.31 (1.20–1.42)	1.44 (1.29–1.61)	0.004
Myocardial infarction (166 out of 10,459)	1.44 (1.23–1.72)	1.66 (1.35–2.17)	0.098
Angina pectoris (158 out of 10,447)	1.17 (1.01–1.36)	1.47 (1.20–1.81)	0.102
Stroke (206 out of 10,482)	1.21 (1.07–1.39)	1.38 (1.17–1.66)	0.063
Men			
Diabetes mellitus (579 out of 9580)^c^	1.21 (1.12–1.31)	1.26 (1.14–1.38)	0.034
Myocardial infarction (550 out of 9540)	1.19 (1.09–1.29)	1.28 (1.14–1.40)	0.044
Angina pectoris (290 out of 9514)	1.08 (0.98–1.21)	1.13 (0.99–1.29)	0.128
Stroke (314 out of 9561)	1.12 (1.01–1.25)	1.13 (1.00–1.29)	0.109

CI: confidence interval; OR: odds ratio.

a Education as linear term, per level decrease.

b *P* value for equality between ORs based on education from Statistics Norway and Tromsø7. The Tromsø Study 2015–16.

c Diabetes mellitus types 1 and 2.

## Discussion

We found that self-reported data on educational level in Tromsø7 achieved very high completeness (⩾97% in all age groups and both sexes) for participants with a college/university education of 4 years or more, and high completeness (67–92% in all age groups and both sexes) for those with a primary educational level. However, low correctness was found for both of these educational levels (29–62% for college/university education ⩾4 years and 48–67% for primary educational level, respectively). Our findings showed substantial agreement (65–71%) in all age groups for men, and moderate to substantial agreement for women (57–64%). Fair weighted kappa values were found in both women (0.41–0.51) and men (0.52–0.59). Educational trends in cardiometabolic diseases were less pronounced when self-reported educational level was used rather than registry-recorded educational level.

The degree of completeness was highest among those with a college/university education of 4 years or more, indicating near-perfect self-reporting. However, completeness among those with primary educational level was slightly lower. Low correctness was found in all age groups in our highest and lowest categories of educational level. There are several possible explanations for this low correctness. First, individuals might consider that they belong in the highest educational category because they have taken courses or programmes that were not necessarily included in a degree. Indeed, it is common in Norway to take work-related continuing education courses, but they do not necessarily culminate in a formal degree. SSB only places individuals in the category of college/university education of 4 years or more if they have completed a Master’s degree or a PhD [11]. In addition, Tromsø7 and SSB measure the educational level differently: SSB asks for the highest completed degree, whereas Tromsø7 asked for the duration of education. Moreover, it has been hypothesised that questionnaire respondents sometimes give answers that are more in line with prevailing social norms than their factual situation [13]. When individuals provide answers they believe to be more socially desirable, rather than revealing their true attitudes, preferences, or beliefs, it is referred to as social desirability bias [14]; it is one of the most common and pervasive sources of bias that affects the validity of survey research findings and might also explain some of the overreporting of the educational level in our study. Previous studies also found that those who claimed to have a degree did not, in fact, have any degree [15, 16].

It is often harder to get a correct answer to questions about education. Some might think they do not have the education they ‘should have’ due to a feeling of social prestige, and therefore report a higher educational level than they actually have [10]. The age group 53–62 years had a higher tendency to overreport their educational level than the youngest age group, while others have found a higher tendency of overreporting among the youngest age group [10]. Second, it is difficult to measure education appropriately, as most societies have complex educational systems that change over time [17, 18]. In Tromsø7, the participants of different age groups have received their education within different school systems, as the Norwegian educational system has been reformed continuously from 1959, which may make it difficult for these participants to report their educational level correctly; for example, the transition from several different degrees with specific Norwegian and Latin titles to Bachelor and Master degrees [19]. SSB has re-classified the educational level of those with what were previously the lowest and middle educational levels due to changes in the Norwegian educational system [10, 20]. Self-reporting of educational level could also be subject to recall bias, particularly among the oldest participants [10, 16].

Finally, overreporting of educational level in questionnaires due to misunderstanding has been reported [21, 22]. It has been suggested that this misunderstanding is linked to the question regarding the duration of education (total years of education versus highest obtained degree) [21, 22], and misclassification can occur when inferring attainment of a degree from years of schooling.

Previous studies observed misreporting of educational level in both sexes, although it was higher among women, which was also the case in our study [15, 23]. Our data suggest that participants from the most affluent households are more likely to overreport their educational level. A previous study found that women who reported having a higher degree also tended to have higher earnings than those who reported their educational level correctly [15]. High-income individuals are more likely than low-income individuals to report their education correctly [23], which is consistent with our findings in the highest household income category.

Knowing the extent of misreporting also has obvious implications for the interpretation of other studies that use educational attainment as an exposure or for descriptive purposes. When education is used as a confounding variable, misclassification may affect the efficiency of adjustment for confounding effects, and thus seriously bias the results [1, 24]. Extensive literature over several decades has reported that people of lower socioeconomic status tend to have a higher prevalence of cardiometabolic diseases [5, 6, 25, 26]. Education is often used as a proxy for socioeconomic status [27], and one purpose of collecting information about education in the Tromsø Study was to use this variable as a proxy for socioeconomic status; thus misreporting may lead to misclassification. This distortion in the association between the exposure and outcome might create a less pronounced educational trend when self-reported educational data are used. Researchers should therefore be aware of the potential shortcomings of using self-reported education compared to administrative records.

### Strengths and limitations

The main strength of this study is the individual complete linkage between a health survey and a national register, using the unique national identification number. The Tromsø Study is a population-based study with a relatively large sample and good representativeness of both women and men. Data on educational level from SSB are based on reports from various educational institutions in Norway and abroad, and we assessed the criterion validity to be reasonably high. This study also has some limitations as the Tromsø Study and SSB measure educational level differently, with Tromsø7 recording years of completed education, and SSB measuring completed education. This might result in low correctness and kappa values in our study. Although the dataset from the SSB had some missing values, the proportion was very low (0.5%) and did not impact the results. Changes in the wording of questionnaires or the addition of extra questions might help future participants to provide their educational level more correctly. For instance, asking for the highest level of education, rather than the number of years of education could improve accuracy.

In conclusion, this study found that data on self-reported educational level in Tromsø7 is adequately complete and correct for research, with fair weighed kappa values in all age groups and both sexes. A considerable proportion of participants, however, did not answer these questions correctly, which can lead to misclassification, and may explain why educational trends in cardiometabolic diseases were less pronounced when using self-reported educational level. We consider our findings to be important for epidemiological research, as they contribute to knowledge on the degree of misclassification and validation of self-reported educational level.

## Supplemental Material

sj-docx-1-sjp-10.1177_14034948221088004 – Supplemental material for Validity of self-reported educational level in the Tromsø Study Click here for additional data file. Supplemental material, sj-docx-1-sjp-10.1177_14034948221088004 for Validity of self-reported educational level in the Tromsø Study by Chi Q Vo, Per-Jostein Samuelsen, Hilde L Sommerseth, Torbjørn Wisløff, Tom Wilsgaard and Anne E Eggen in Scandinavian Journal of Public Health
