# Supplementary material for: Validity of self-reported educational level in the Tromsø Study
Source: Scand J Public Health. 2022 May 20;51(7):1061–8. doi: 10.1177/14034948221088004 (PMC10599084; doi:10.1177/14034948221088004)
Supplement: sj-docx-1-sjp-10.1177_14034948221088004 – Supplemental material for Validity of self-reported educational level in the Tromsø Study [file sj-docx-1-sjp-10.1177_14034948221088004.docx]

**Supplementary data**

Supplement to: Vo CQ, et al. Validity of self-reported educational level in the Tromsø Study.


**Table 1.** Sex-specific cross tabulation of self-reported and Statistics Norway-recorded educational level by age. The Tromsø Study 2015-2016. Bolded numbers reflect positive predictive value.

| Statistics Norway | | | | | |  |  |
| --- | --- | --- | --- | --- | --- | --- | --- |
| Tromsø7 | **Age group**  **Women** | Primary education,  n (%) | Upper secondary education,  n (%) | College/university <4 years​,  n (%) | College/university **≥**4 years​,  n (%) | Agreement (%) | Weighted kappa |
|  | **40-52 years** |  |  |  |  | 64.4 | 0.51 |
|  | Primary education | **258 (66.8)** | 125 (32.4) | 3 (0.8) | 0 (0.0) |  |  |
|  | Upper secondary education | 100 (9.3) | **921 (86.2)** | 47 (4.4) | 1 (0.1) |  |  |
|  | College/university <4 years​ | 20 (2.1) | 190 (20.0) | **733 (77.2)** | 7 (0.7) |  |  |
|  | College/university **≥**4 years​ | 6 (0.3) | 16 (0.8) | 1040 (52.9) | **905 (46.0)** |  |  |
|  | **53-62 years** |  |  |  |  | 60.8 | 0.48 |
|  | Primary education | **403 (62.8)** | 234 (36.4) | 5 (0.8) | 0 (0.0) |  |  |
|  | Upper secondary education | 127 (14.5) | **719 (82.2)** | 28 (3.2) | 1 (0.1) |  |  |
|  | College/university <4 years​ | 36 (6.4) | 147 (26.3) | **371 (66.4)** | 5 (0.9) |  |  |
|  | College/university **≥**4 years​ | 6 (0.6) | 15 (1.5) | 598 (60.4) | **372 (37.5)** |  |  |
|  | **63-99 years** |  |  |  |  | 56.7 | 0.41 |
|  | Primary education | **759 (48.4)** | 803 (51.2) | 7 (0.4) | 0 (0.0) |  |  |
|  | Upper secondary education | 55 (6.8) | **709 (88.1**) | 40 (5.0) | 1 (0.1) |  |  |
|  | College/university <4 years​ | 6 (1.5) | 117 (29.0) | **276 (68.3)** | 5 (1.2) |  |  |
|  | College/university **≥**4 years​ | 5 (0.8) | 7 (1.1) | 420 (69.0) | **177 (29.1)** |  |  |

Percentages calculated to equal 100% in row.

**Table 2.** Sex-specific cross tabulation of self-reported and Statistics Norway-recorded educational level by age. The Tromsø Study 2015-2016. Bolded numbers reflect positive predictive value.

| Statistics Norway | | | | | |  |  |
| --- | --- | --- | --- | --- | --- | --- | --- |
| Tromsø7 | **Age group**  **Men** | Primary education,  n (%) | Upper secondary education,  n (%) | College/university <4 years​,  n (%) | College/university **≥**4 years​,  n (%) | Agreement (%) | Weighted kappa |
|  | **40-52 years** |  |  |  |  | 70.6 | 0.59 |
|  | Primary education | **337 (65.8)** | 175 (34.2) | 0 (0.0) | 0 (0.0) |  |  |
|  | Upper secondary education | 101 (8.1) | **1083 (87.3)** | 54 (4.4) | 3 (0.2) |  |  |
|  | College/university <4 years​ | 23 (2.7) | 274 (32.5) | **543 (64.3)** | 4 (0.5) |  |  |
|  | College/university **≥**4 years​ | 5 (0.4) | 38 (3.0) | 461 (36.4) | **764 (60.2)** |  |  |
|  | **53-62 years** |  |  |  |  | 66.9 | 0.54 |
|  | Primary education | **364 (61.4)** | 227 (38.2) | 1 (0.2) | 1 (0.2) |  |  |
|  | Upper secondary education | 89 (10.5) | **730 (86.1)** | 26 (3.1) | 3 (0.3) |  |  |
|  | College/university <4 years​ | 28 (4.7) | 239 (40.2) | **321 (53.9)** | 7 (1.2) |  |  |
|  | College/university **≥**4 years​ | 1 (0.2) | 19 (2.9) | 248 (38.4) | **378 (58.5)** |  |  |
|  | **63-99 years** |  |  |  |  | 64.9 | 0.52 |
|  | Primary education | **545 (51.5)** | 513 (48.5) | 0 (0.0) | 0 (0.0) |  |  |
|  | Upper secondary education | 45 (5.0) | **797 (88.6**) | 56 (6.2) | 2 (0.2) |  |  |
|  | College/university <4 years​ | 9 (1.4) | 259 (40.3) | **363 (56.4)** | 12 (1.9) |  |  |
|  | College/university **≥**4 years​ | 1 (0.2) | 34 (5.3) | 208 (32.4) | **398 (62.1)** |  |  |

Percentages calculated to equal 100% in row.

**Table 3.** Distribution of self-reported and Statistics Norway-recorded educational level by age and sex. The Tromsø Study 2015-2016.

| **Women** (n = 10 826) **Men** (n = 9789) | | | | |
| --- | --- | --- | --- | --- |
| **Age group** | **Tromsø7**  n (%) | **Statistics Norway**  n (%) | **Tromsø7**  n (%) | **Statistics Norway**  n (%) |
| **40-52 years** | |  |  |  |
| Primary education | 386 (8.8) | 384 (8.8) | 512 (12.3) | 466 (12.1) |
| Upper secondary education | 1069 (24.5) | 1251 (28.6) | 1241 (32.1) | 1570 (40.6) |
| College/university <4 years​ | 950 (21.7) | 1823 (41.7) | 844 (21.8) | 1058 (27.4) |
| College/university **≥**4 years​ | 1967 (45.0) | 913 (20.9) | 1268 (32.8) | 771 (19.9) |
| **53-62 years** | |  |  |  |
| Primary education | 642 (20.9) | 572 (18.6) | 593 (22.1) | 482 (18.0) |
| Upper secondary education | 875 (28.5) | 1115 (36.4) | 848 (31.6) | 1215 (45.3) |
| College/university <4 years​ | 559 (18.2) | 1002 (32.7) | 595 (22.2) | 596 (22.2) |
| College/university **≥**4 years​ | 991 (32.3) | 378 (12.3) | 646 (24.1) | 389 (14.5) |
| **63-99 years** |  |  |  |  |
| Primary education | 1569 (46.3) | 825 (24.4) | 1058 (32.6) | 600 (18.5) |
| Upper secondary education | 805 (23.8) | 1636 (48.3) | 900 (27.8) | 1630 (49.4) |
| College/university <4 years​ | 404 (11.9) | 743 (21.9) | 643 (19.8) | 627 (19.3) |
| College/university **≥**4 years​ | 609 (18.0) | 183 (5.4) | 641 (19.8) | 412 (12.7) |
